# Strained Monolayer MoTe_2_ as a Photon Absorber in the Telecom Range

**DOI:** 10.3390/nano13202740

**Published:** 2023-10-10

**Authors:** Muhammad Sufyan Ramzan, Caterina Cocchi

**Affiliations:** 1 Institut für Physik, Carl von Ossietzky Universität, 26129 Oldenburg, Germany; 2 Center for Nanoscale Dynamics (CeNaD), Carl von Ossietzky Universität, 26129 Oldenburg, Germany

**Keywords:** monolayer MoTe_2_, straintronics, photon absorber, first-principle calculations

## Abstract

To achieve the atomistic control of two-dimensional materials for emerging technological applications, such as valleytronics, spintronics, and single-photon emission, it is of paramount importance to gain an in-depth understanding of their structure–property relationships. In this work, we present a systematic analysis, carried out in the framework of density-functional theory, on the influence of uniaxial strain on the electronic and optical properties of monolayer MoTe_2_. By spanning a ±10% range of deformation along the armchair and zigzag direction of the two-dimensional sheet, we inspect how the fundamental gap, the dispersion of the bands, the frontier states, and the charge distribution are affected by strain. Under tensile strain, the system remains a semiconductor but a direct-to-indirect band gap transition occurs above 7%. Compressive strain, instead, is highly direction-selective. When it is applied along the armchair edge, the material remains a semiconductor, while along the zigzag direction a semiconductor-to-metal transition happens above 8%. The characteristics of the fundamental gap and wave function distribution are also largely dependent on the strain direction, as demonstrated by a thorough analysis of the band structure and of the charge density. Additional ab initio calculations based on many-body perturbation theory confirm the ability of strained MoTe_2_ to absorb radiation in the telecom range, thus suggesting the application of this material as a photon absorber upon suitable strain modulation.

## 1. Introduction

The successful exfoliation of graphene and the discovery of its astonishing electronic properties [1] have directed the interest of the scientific community towards two-dimensional (2D) materials since the beginning of the 21st century. To date, several classes of 2D materials have been experimentally realized [2,3,4,5,6,7,8,9,10,11] and many more have been theoretically predicted [11,12,13,14]. Among them, transition metal dichalcogenides (TMDCs) with chemical formula MX_2_ (where M = Mo, W, and X = S, Se, Te) are semiconductors that have attracted significant attention owing to their unique opto-electronic properties [7,9,15,16,17,18,19]. The flexibility of these materials enables the tuning of their features by structural modulation. In contrast to electric control [20,21] or neutron-transmutation doping [22], which are purposefully applied via external gating, mechanical deformation can occur spontaneously upon substrate deposition [23] and in the formation of van der Waals heterostructures [24,25,26,27]. For example, strain has been largely exploited to modify the electronic and optical response of TMDCs [20,21,22,23,24,25,26,27,28]. In particular, for several monolayers (ML) belonging to this family, strain engineering has been employed to achieve an indirect-to-direct band gap transition, to enhance thermal conductivity, or to induce a semiconductor-to-(semi-)metal transition [21,22,23,24,28,29,30]. For multilayer systems, strain engineering was also used to turn the intrinsic indirect band gap into a direct one [20,28]. Computational modeling has provided substantial contributions to this field of research by predicting and rationalizing the behavior of strained TMDCs in a systematic way [30,31,32] and by contributing to the understanding of their electronic and vibrational responses [30,33,34]. Relevant efforts have also been devoted to assessing the impact of strain on the excitons that dominate the optical spectra of these materials [35,36]. 

Despite this wealth of findings, the knowledge about the effects of strain on the intrinsic characteristics of TMDCs is still incomplete. In particular, questions regarding the interplay between the macroscopic deformations undergone by the strained material and the microscopic variations in its atomic structure and their influence on electronic and optical responses are still unanswered. The issues are relevant not only on a fundamental level, but are also crucial for fabricating nano- and heterostructures with properties targeting the desired application. Indeed, strain effects are ubiquitous when different monolayers (MLs) are stacked to form a hetero-multilayer [37,38,39]; they emerge from variations in the lattice parameters of the 2D systems as well as from the influence of a substrate [40]. The case of MoTe_2_ is particularly relevant in this context. Due to its low band gap (1.1 eV) [41] in the near-infrared region and its predicted hyperbolic response to radiation [42], this material has recently gained interest as a potential candidate for applications in integrated optoelectronics [43], including light-emitting devices and near-infrared photodetectors [44,45], photoconductors [46], and spin-valley quantum gates [47]. The nonlinear optical response of this TMDC has been recently explored as well [48,49]. Moreover, in recent years, MoTe_2_ has been largely explored in the field of telecommunications due to its high photo-response in the relevant frequency range [29,50,51,52]. Notably, strain has been identified as an effective means to fine-tune the characteristics of the system and optimize device performance [34,50,52]. These late experimental efforts call for a deeper understanding of the fundamental properties of strained MoTe_2_ as a robust candidate in this field of application.

In this paper, we present a systematic analysis of the electronic structure and optical response of ML MoTe_2_ under the influence of uniaxial strain obtained from state-of-the-art first-principles calculations based on density-functional theory (DFT) and many-body perturbation theory (MBPT). Our goal is to disclose the interplay between the macroscopic deformations of the strained lattice and the microscopic variations induced in the electronic structure of the material. For this purpose, we explore a wide range of strain up to ±10% of the lattice parameters. To perform this analysis, we apply strain along the two orthogonal directions of ML MoTe_2_, commonly defined as *armchair* and *zigzag*. We discuss how the electronic characteristics of the material, such as the nature and the size of the band gap, are influenced by the amount of the applied strain and by its direction. To do so, we inspect the band structure and we thoroughly analyze the charge-density distribution as a function of the strain. Furthermore, we compute the optical properties of MoTe_2_ at selected amounts of strain and quantitatively assess the energy of the lowest absorption maximum. Our findings confirm that this material can indeed act as a photon absorber in the telecom range (0.8–1.2 eV) upon appropriate strain modulation.

## 2. Methods

The results presented in this work from DFT [53] and MBPT [54] (*GW* approximation and Bethe–Salpeter equation) are obtained using the Vienna ab initio simulation (VASP) code [48]. In DFT, the interactions between electrons and nuclei in the Kohn–Sham (KS) equations [49] are described using the projector-augmented wave (PAW) method [55]. Structural optimization and electronic structure calculations are performed within the generalized gradient approximation for the exchange correlational potential, as proposed by Perdew, Burke, and Ernzerhof (PBE) [56], augmented by Grimme’s DFT−D3 correction [57] to account for dispersive interactions. Spin–orbit coupling (SOC) is included in all calculations. Given the known flaws of PBE in the quantitative prediction of the electronic structure of semiconductors and in particular of their band gaps, additional calculations are performed using the range-separated hybrid functional HSE06 [58,59] to check that the trends provided by the PBE results are correct. A 12 × 12 × 1 **k**-point mesh with a 500 eV plane wave energy cutoff is used to relax the lattice vectors and the atomic positions in the unit cell. Convergence thresholds of 1 × 10^−8^ eV and 10 meV Å^−1^ are adopted for electronic self-consistency and for minimizing the residual interatomic forces, respectively. The structure of ML MoTe_2_ is extracted from the bulk 2H phase of the material. A vacuum layer of 15 Å is inserted along the non-periodic vector to isolate the ML from its periodic images. In the MBPT calculations, quasiparticle energies are obtained from the single-shot *G_0_W_0_* approach [60] applied on top of the DFT electronic structure, and optical properties are subsequently calculated by solving the Bethe–Salpeter equation (BSE) [61] on top of the *GW*-corrected energy eigenvalues. For *GW* and BSE calculations, including spin–orbit coupling, a 12 × 12 × 1 **k**-point mesh and 300 bands in total are accounted for. The 12 highest valence bands and the 30 lowest conduction bands are included in the solution of the BSE.

**Figure 1:**
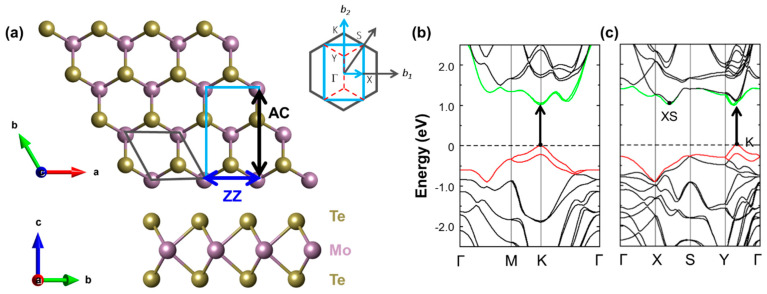
(**a**) Top and side view of a 4 × 4 MoTe_2_ crystal structure with the hexagonal (orthorhombic) unit cell marked in grey (light blue). The armchair (AC; black) and zigzag (ZZ; blue) directions are marked arrows. The Brillouin zones corresponding to both hexagonal and orthorhombic unit cells are shown in the inset with the same color code; only the high-symmetry points of the orthorhombic representation are displayed. Electronic band structures of MoTe_2_ calculated at the PBE level of theory (**b**) in the hexagonal representation and (**c**) in the orthorhombic one. The band gap is marked with a black arrow. The Fermi-level (horizontal dashed line) is aligned to the VBM and set to zero.

To simulate uniaxial strain along the armchair (AC) and zigzag (ZZ) directions of the ML, we adopt the orthorhombic representation of the unit cell (see Figure 1a). Strain is applied by modifying the lattice parameters of the considered orthorhombic unit cell of MoTe_2_ ranging from 0% (unstrained material) up to ±10%, where positive (negative) values indicate tensile (compressive) deformation. The resulting atomic positions are subsequently optimized to minimize the forces between them according to the procedure and the thresholds reported above. In the adopted representation, the Brillouin zone is also orthorhombic, and the band structure of the system is represented along the path connecting the high-symmetry points of this crystal structure (see inset in Figure 1a). While a transformation into the hexagonal cell could be performed by means of band unfolding techniques [62,63,64], hereafter, we purposely stick to the orthorhombic representation to relate the electronic structure of the strained material with the directions of application of strain. The analysis of the microscopic charge distribution is performed with the aid of Bader charge analysis [65,66,67]. To check the dynamical stability of strained MoTe_2_ ML, a 2 × 3 × 1 orthogonal supercell was adopted to calculate the phonon dispersion spectrum, employing the finite displacement method as implemented in Phonopy [68]. Crystal structures and wave function plots are visualized using VESTA [69], and for the band structures, the post-processing code VASPKIT is used [70].

## 3. Results and Discussion

### 3.1. Unstrained MoTe_2_: Structural and Electronic Properties

As the first step of our analysis, we optimize the pristine MoTe_2_ ML, initially considering the conventional hexagonal representation for comparison with the literature. The obtained lattice constant (*a* = *b*), equal to 3.52 Å, is in good agreement with the results of earlier works (*a* = *b* = 3.55 Å) performed at the same level of theory [71,72,73]. We calculate its band structure and find the expected features (see Figure 1b): both the valence band maximum (VBM) and the conduction band minimum (CBM) are located at the high-symmetry point K. The highest occupied band undergoes a spin–orbit splitting of about 214 meV in the vicinity of the VBM, while in the lowest conduction band, the sub-band separation is on the order of 30 meV. The band gap is equal to 1.0 eV, in agreement with results from the literature obtained with the same approximations adopted here [71,72,73]. It is worth noting that the band gap obtained from DFT with the PBE functional is in remarkably good agreement with experimental values from optical measurements [41]: this coincidence is simply due to error cancellation. 

The electronic properties obtained in the conventional, hexagonal cell of ML MoTe_2_ are visible in the band structure computed in the orthorhombic representation. For this unit cell, marked in Figure 1a by a light blue rectangle, we obtain lattice constants of 3.50 Å and 6.07 Å along the ZZ and AC directions, respectively. Due to the absence of a direct equivalent of the K point, the VBM and CBM fall between the points Y and Γ (see Figure 1c). We denote this point as K. Moreover, we identify in Figure 1c the point of an additional valley in the conduction band, labeled XS as it is along the path connecting the high-symmetry points X and S. As discussed in the following, this point becomes relevant in the electronic structure of strained MoTe_2_.

### 3.2. Electronic Structure of Uniaxially Strained MoTe_2_

In the next step of our analysis, we inspect the electronic structure of MoTe_2_ under different amounts of tensile and compressive strain applied along the armchair and zigzag directions. We explore strain within the range [−10%, 10%] of the unit cell parameters with steps of 1%. Strain in the order of 1–3% is known to promote the transition from the semiconducting 2H phase of MoTe_2_ to the semimetallic 1T’ one by reducing the corresponding barrier [72,74]; in this process, thermodynamic and kinetic effects have been demonstrated to play an important role. Larger values of global strain up to |10|% are seldomly explored experimentally [75]. However, investigating the corresponding structures from first principles is expected to provide invaluable insights into the intrinsic properties of ML MoTe_2_. Moreover, we recall that curved TMDC nanostructures, such as nanoripples or nanobubbles, may host very high levels of local strain [70]. We checked the stability of the considered structures under strain by computing the phonon dispersion (see Figures S1 and S2) and found dynamical instability only for −10% strain in the ZZ direction. Further discussion on this point is provided in the Supporting Information.

**Figure 2:**
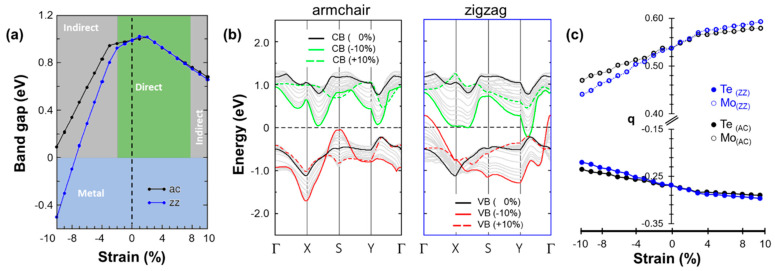
(**a**) Electronic band gap of MoTe_2_ computed from PBE as a function of strain applied along the armchair (black) and zigzag (blue) directions. The nature of the gap is indicated by colored areas: green, grey, and light blue stand for direct, indirect, and zero gap, respectively. (**b**) Energy dispersion of the valleys in the valence (red) and conduction regions (green) under different values of tensile (dashed) and compressive (solid) strain. The highest valence and the lowest conduction band of the unstrained ML are shown for reference by black solid lines. (**c**) Average partial charge per atom q for different values of strain applied in the armchair (black) and zigzag (blue) direction.

From the results summarized in Figure 2a, we find that under tensile strain up to 8% along either the ZZ or AC direction, the band gap remains direct at the K point; for larger values of strain, it turns into an indirect gap. The amplitude of the band gap is identical along the two directions except for 1% strain, where the deviation is of the order of 2 meV, and strain larger than 8%, where the values differ by up to 23 meV. Notably, for 1% strain the band gap is larger along the ZZ direction, while for strain > 8% the opposite is true. We additionally checked that the range-separated hybrid functional HSE06 gives an analogous trend for the band gaps (see Figure S3). The trend reported in Figure 2a is in general agreement with previous work based on DFT and investigating the effects of uniaxial strain in ML MoTe_2_ [31]. 

Turning now to the band structures (see Figure 2b), we notice that even though the dispersion of valence valleys varies substantially, the position of the VBM changes insignificantly in energy and momentum. Compared to the result obtained for the unstrained ML, the dispersion of valence valleys at the K point decreases (increases) when strained along the AC (ZZ) direction. In both cases, the CBM changes significantly and the conduction valleys move downward in energy. In addition, increasing strain along the AC (ZZ) direction shifts these valleys towards the high-symmetry point Γ (Y), causing a direct-to-indirect band gap transition. The full band structure under tensile and compressive strain along the armchair (zigzag) direction is reported in Figures S4 and S5 (Figures S6 and S7), respectively.

Compressive strain has a much more direction-selective impact on the material, while in general the band gap is reduced by increasing strain along both the zigzag and armchair directions; in the former case, ML MoTe_2_ undergoes a semiconductor-to-metal transition for strain values larger in magnitude than −7% (see Figure 2a). At smaller values of strain, between 0 and −3%, a direct-to-indirect band gap transition occurs in both strain directions, AC and ZZ. The system subject to −3% strain has a smaller band gap when the deformation is applied along the ZZ edge than along the AC one. Valence and conduction valleys change significantly under compressive strain along the AC direction (see Figure 2b). The VBM shifts to the S point when the strain is larger than −3%, causing the above-mentioned direct-to-indirect band gap transition. Moreover, the valley at the S point increases in energy compared to the VBM of the unstrained ML. On the other hand, the conduction valleys gradually move downward in energy with increasing strain without a significant change in their dispersion. Remarkably, the value of the direct gap remains almost unaltered, around 750 meV, at higher values of strain (>5%) due to the sizeable increase in the VBM at S with the concomitant decrease in both valleys in the conduction band (see Figure 2b). At very large compressive strains (>|−8|%), the CBM shifts to the point labeled XS. The change in shape of the valleys and their energy shift in both the valence and conduction regions are much more significant under compressive strain in the ZZ direction. In the valence band, the valley at the Γ point undergoes a significant increase in energy and crosses the Fermi-level when the strain is larger than −7%. On the other hand, at the same threshold, the CBM at the K point shifts downward and crosses the Fermi level, turning the system into a metal. 

### 3.3. Charge-Density Distribution and Structure–Property Relationship

For an all-around characterization of the electronic structure of strained ML MoTe_2_, we additionally performed a Bader charge analysis. In the unstrained system simulated in the orthorhombic unit cell, the Mo (Te) atom has an average charge of +0.51e (−0.26e) (see Figure 2c), indicating a non-negligible degree of polarity in the covalent Mo-Te bonds. Under small values of strain (±3%), the average bond polarization remains unchanged regardless of the direction of deformation. However, the trends start to diverge as the strain magnitude is further increased. Under tensile strain, when the change in the electronic structure is moderate, the values of the partial charge *q* are rather similar on both Mo and Te atoms belonging to strained systems along AC and ZZ directions. In general, increasing tensile strain enhances the Mo-Te bond polarization: for the system with 10% strain, the charge on the Mo atom increases to about +0.6e, corresponding to a relative increment of >16%. Conversely, compressive strain generally reduces the polarization of the Mo-Te bond. Notably, this trend is direction-selective: for 10% strain along the zigzag (armchair) direction, the charge on the Mo atom decreases to +0.44e (+0.47e), corresponding to a drop of almost 14% (8%). 

**Figure 3:**
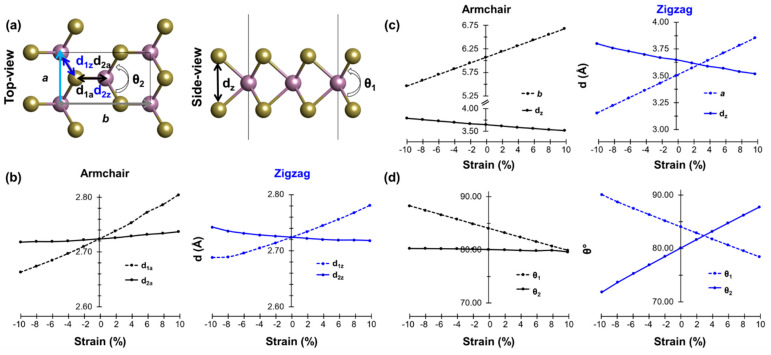
(**a**) Top and side view of ML MoTe_2_ (unit cell boundaries highlighted by the thin black rectangle) with the strain along the armchair (zigzag) direction marked with a grey (light blue) arrow. Variation (**b**) in the lattice parameters and in the vertical Te-Te distance, and (**c**) in the bond lengths labeled in panel (**a**,**d**) of the in-plane and out-of-plane Te-Mo-Te angles (θ_1_ and θ_2_, respectively) as a function of strain applied along the armchair direction (left panels) and the zigzag direction (right panels).

The results of this analysis call for a deeper understanding of the connection between the strain and microscopic characteristics of the electronic structure of the material. To this end, we compare the relationship between macroscopic properties such as the elongation/contraction of the lattice vectors and the microscopic response of ML MoTe_2_ in terms of changes in the bond lengths and angles. To do so, we focus on the following structural parameters (see Figure 3 top panel): With d_1a_ (d_1z_) and d_2a_ (d_2z_), we indicate the bond lengths along the armchair (zigzag) direction, and with θ_1_ and θ_2_ we indicate the Te-Mo-Te angles in the in-plane and out-of-plane directions, respectively. Finally, we mark as d_z_ the vertical distance between the two Te atoms bound to the same Mo atom. Again, we monitor the corresponding trends for structures under tensile and compressive strain along the AC and ZZ directions.

The application of strain along the AC direction leads to a substantial variation in the parallel Mo-Te bond length (d_1a_) and a negligible change in d_2a_. Along the ZZ direction, on the other hand, the variation in d_2z_ is much smaller compared to that in d_z1_. These trends are rather intuitive: a deformation in a certain direction primarily impacts the chemical bonds in the same direction. A quantitative inspection of the plots in Figure 3b reveals a larger variation in d_1a_ compared to d_1z_. This behavior can be understood by considering that d_1a_ is parallel to the AC direction while d_1z_ forms an angle of about 29° with the ZZ direction. Moving now to the analysis of d_z_, namely the vertical separation between two Te atoms bound to the same Mo, we notice a variation of the order of 1 Å over the spanned range of strain (see Figure 3c). Specifically, the ML becomes “thinner” upon tensile strain and vice versa. In Figure 3c, the variations in the lattice parameters *a* and *b* are shown upon the application of strain along the ZZ and AC directions, respectively. 

Finally, we comment on the behavior of angles θ_1_ and θ_2_ under strain, which exhibit substantially different trends (see Figure 3d). Reflecting the variation in d_z_ upon strain, the corresponding Te-Mo-Te angle, θ_1_, is insensitive to the application of strain along the AC or ZZ direction. In both cases, the amplitude of the angle increases upon compressive strain and decreases upon tensile deformation with respect to the pristine ML. In contrast, the behavior of θ_2_ is strongly dependent on the direction of the applied strain. Along the AC edge, it stays almost constant independently of the magnitude and the type of strain. Conversely, along ZZ, θ_2_ increases (decreases) with tensile (compressive) strain by about 10° compared to the reference value in the unstrained structure. Once again, this behavior is consistent with physical intuition: straining the material along the ZZ direction directly impacts the angle θ_2_, while a deformation along AC expectedly has no influence on it.

**Figure 4:**
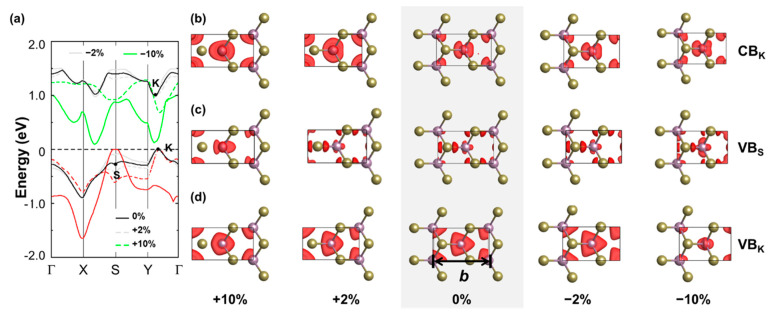
(**a**) Dispersion of the highest occupied (red) and lowest unoccupied (green) bands of MoTe_2_ under tensile (dashed lines) and compressive strain (solid lines) with magnitude |2%| (gray) and |10%| (green) along the armchair direction. The bands of the unstrained ML are shown in black for reference. The Fermi level (horizontal dashed line) is aligned to the VBM and set to zero. Probability densities of the KS states of the (**b**) conduction band (CB) at the high-symmetry point K, (**c**) of the valence band (VB) at the high-symmetry point S and (**d**) of the VB at the high-symmetry point K. For all of the wave function plots, an iso-value of 0.0035 is adopted.

Next, we examine the effect of strain in the real-space representation of the probability density associated with the KS wave functions at selected high-symmetry points. We start from strain applied along the AC direction (see Figure 4), and we inspect the wave functions of the highest valence at the high-symmetry points S and K (VBM), as well as of the CBM at K. For clarity, we focus on deformations with moderate (±2%) and high (±10%) values of strain, which are appropriate to support the following discussion. In fact, systems with ±4% (±8%) values of strain behave like those of ±2% (±10%). The character of the wave function for the unstrained material is shown for comparison and highlighted by the gray background. The frontier states at K are localized on the Mo atom (*d* orbital), while the valence band at S is spread along the Mo-Te bond. Upon compressive strain, variations in the wave function character are hardly perceivable from visual inspection, even for high values of strain (−10%). A notable exception is given by the wave function of the valence band at K under −10% strain. In this configuration, the energetic order of the states changes with respect to that of pristine ML MoTe_2_, and the highest valence state at K corresponds to the second highest one in the pristine material (see Figure 2b). Considering now the wave functions in the ML undergoing tensile strain along the AC direction, we notice more remarkable differences with respect to the unstrained case, which may be related to the shift of the valleys in these configurations in energy and along the k-direction (see Figure 2b). For the examined conduction state at K, even at a moderate amplitude of tensile strain (2%), the character of the wave function changes with respect to the pristine material, again as a consequence of the reordering of the electronic states at the frontier (Figure 4a). In the two occupied states depicted in Figure 4b,c, a change in character is noticed only at S for the largest strain. At K, the wave function is unaffected except for a perturbation of its spatial distribution, which becomes more anisotropic due to the deformation.

**Figure 5:**
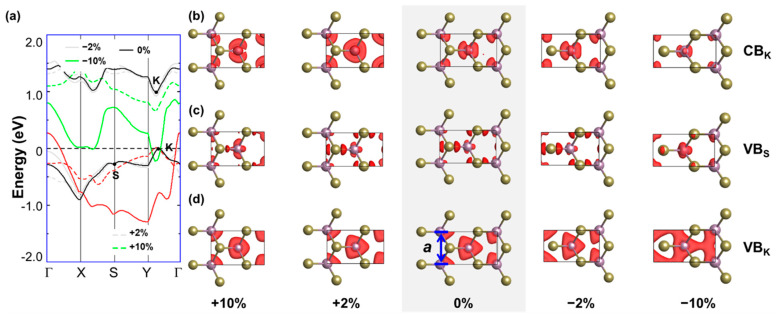
(**a**) Dispersion of the highest occupied (red) and lowest unoccupied (green) bands of MoTe_2_ under tensile (dashed lines) and compressive strain (solid lines) with magnitude |2%| (gray) and |10%| (green) along the zigzag direction. The bands of the unstrained ML are shown in black for reference. The Fermi level (horizontal dashed line) is aligned to the VBM and set to zero. Probability densities of the KS states of the (**b**) conduction band (CB) at the high-symmetry point K, (**c**) of the valence band (VB) at the high-symmetry point S and (**d**) of the VB at the high-symmetry point K. For all the wave function plots, an iso-value of 0.0035 is adopted, except for VB_s_ under −10% strain, for which an iso-value of 0.00035 is used.

Moving to the ML strained along the ZZ direction, we notice similar trends (see Figure 5). However, in this case, deformations do not impact the Mo-Te bond along the longer lattice parameter of the orthorhombic unit cell, but rather the Te-Mo-Te angle (θ_2_, see Figure 3). As a result, under a compressive strain of −10%, the spread of the wave function of the CB at K is almost entirely along the AC direction. A similar behavior under compressive strain is also seen for the valence band at S, with the result at −10% showing that the probability density of this state increases around the metal atom. On the other hand, due to its intrinsic character, the valence state at K becomes substantially more delocalized under compressive strain: in the extreme case (−10%), we see in Figure 5c a continuous charge distribution connecting the Mo atoms. Turning now to tensile strain, the opposite trends are noticed for the considered occupied states. They preserve their character and their spatial distribution becomes more isotropic, in opposition to their counterparts obtained upon tensile AC strain. This behavior can be understood by considering that an elongation of the orthorhombic unit cell along the short lattice parameter makes the unit cell itself less anisotropic, with evident consequences on the single electronic wave functions. On the other hand, the CBM at K assumes a different character upon ZZ tensile strain. Additionally, in this case, the overall spatial distribution of the wave function becomes increasingly isotropic with increasing strain magnitudes. The exact position of CBM and VBM may vary slightly depending on the **k**-point sampling of the Brillouin zone. We checked that by doubling the density of the **k**-grid between the high-symmetry points, we do not change the overall trend (see Figure S8).

### 3.4. Photon Absorption in the Telecom Range

To qualitatively analyze the relationship between uniaxial strain and photon absorption in the telecom range, we calculate the optical gap of ML MoTe_2_ with selected values of compressive and tensile strain by solving BSE on top of the *GW*-corrected eigenvalues. With this approach, we are able to account for electron–electron correlations (*GW*), as well as for electron–hole interactions (BSE), which both play a crucial role in determining the optical properties of MoTe_2_ [42,76,77,78] and of TMDCs in general [19]. Since we are interested in assessing the ability of the material to act as an absorber in the region of 0.82–1.18 eV, we focus specifically on the lowest energy absorption maximum (details in Table S1). In the unstrained case considered for reference, we find a *GW* gap of 1.65 eV, in very good agreement with earlier reports [42,77,78,79], and a lowest energy excitation of 1.28 eV. Assuming for this value an uncertainty of the order of 100 meV due to numerical convergence and missing phonon screening [80], we again find good agreement with calculated values available in the literature for unstrained ML MoTe_2_ [42,77,78,79]. 

**Figure 6:**
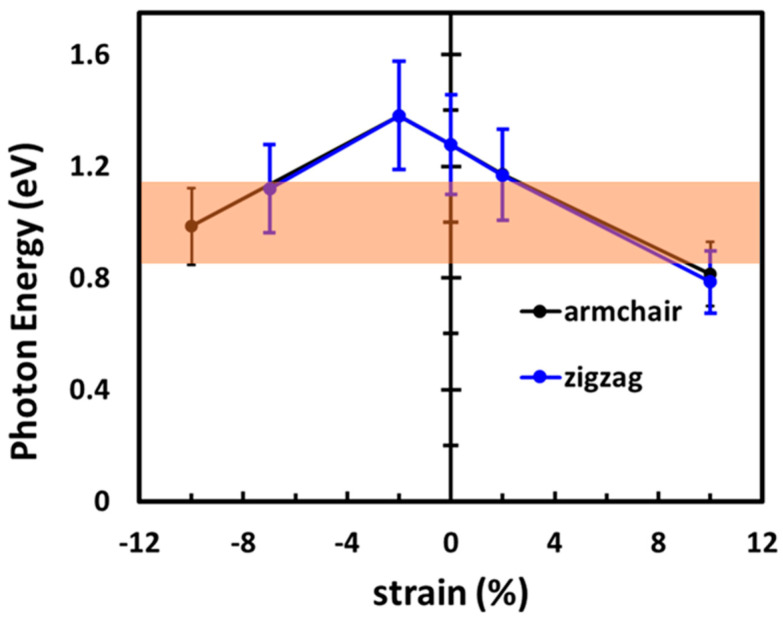
Optical absorption onset calculated for ML MoTe_2_ under selected values of strain along the armchair (black) and zigzag (blue) directions. The error bars cover the uncertainty in the calculations. The orange rectangle highlights the region of interest for telecom applications (0.82–1.18 eV).

We visualize the photon absorption trends of strained ML MoTe_2_ in Figure 6, highlighting in orange the telecom range (0.82–1.18 eV). Due to the metallicity of ML MoTe_2_ for large elongations in the ZZ direction, in this case, the first absorption maximum was calculated for compressive strain values of −2% and −7%, while along AC we explore the whole considered range until −10%. Upon tensile strain, the representative configurations with 2% and 10% strain are explored. The results reported in Figure 6 follow the qualitative trends of the band gaps displayed in Figure 2a: the absorption onset is highest in energy upon −2% compressive strain in either direction. The lowest energy excitation falls into the telecom range for values of compressive strain larger than |−5|%. For the extreme deformation with −10% strain along the AC direction, we find the absorption onset, including its uncertainty, entirely within the desired window. Under compressive strain, for which the system remains semiconducting regardless of the strain direction, photon absorption in the telecom window is predicted over a large range of deformation, ranging from 2% up to 10%. The absorption onset of unstrained MoTe_2_ lies at the upper boundary of the region of interest, suggesting that the presence of defects that naturally occur in actual samples [81,82] or the deposition of the ML on a screening substrate [83] may downshift the absorption onset to the necessary amount (in the order of a few tens of meV) to fit in the desired window.

## 4. Discussion

The analysis presented above illustrates the profound impact of uniaxial strain on the electronic and optical properties of monolayer MoTe_2_, making it a promising material for the absorption of telecom radiation. Changes in the band structure do not only involve the nature of the band gap and its size, as previously discussed in the literature [21,23,26,31,33,84], but they also affect the system on a microscopic level by modifying the dispersion of the valleys as well as the energy and the location of the frontier states in **k**-space. The implications of this tunability in the emerging fields of spintronics [85], valleytronics [86,87], and single-photon emission are evident. The direction of application of strain does not play a role in this regard, thus reducing the number of degrees of freedom to be handled experimentally. On the other hand, by controlling the amount and the direction of the uniaxial strain, it is possible to control the valence and conduction band extrema. Modifications in the electronic structure occur already at moderate strain values (a few percent of the lattice parameters of the pristine material), which can be generated by external mechanical manipulations [80] or through the interaction of the ML with a substrate [50,88] or another 2D sheet [39,89]. In such a scenario, and in particular in the case of non-negligible electronic interactions among the materials in the sample, the perturbation induced by strain on the electronic states of MoTe_2_ can lead to a substantial impact on the electronic and optical response of the whole system. The strain-induced variation in the electronic structure identified by our study concerns both the energy of the single-particle levels (the VBM and CBM, which change character under strain, indicate a reordering of the bands) as well as the spatial distribution of the corresponding wave functions. The latter, in particular, can influence hybridization with other species, including interfaces with organic molecules [64], and also optical selection rules [36,90] and vibrational responses [30]. 

The absorption onset calculated for strained ML MoTe_2_ falls within the telecom window (0.82–1.18 eV) for a large range of deformations, both compressive and tensile. Following the same qualitative trend as the fundamental gap, the optical gap including excitonic effects exhibits the largest values, with values of compressive strain around −2%. A larger compressive strain is expected to enhance the excellent absorption properties of the material in the telecom band. Tensile deformations, on the other hand, are expected to make ML MoTe_2_ a good photon absorber in the region of interest for medium values of strain. It is worth noting that the direction of uniaxial strain does not play a major role in this process except when it makes the material metallic, as in the case of large compressive strain in the zigzag direction. 

## 5. Conclusions and Outlook

In conclusion, we have presented a systematic analysis of the influence of uniaxial strain, both tensile and compressive, on the electronic and optical properties of monolayer MoTe2, and have selectively applied such strain along the armchair and zigzag directions. We have identified the |2|%–|5|% range of strain (both compressive and tensile) as the one favoring the application of this material as a photon absorber in the telecom window (0.82–1.18 eV). We have analyzed the variations induced in the character and in the size of the fundamental gap, and we have monitored how the charge distribution and the single-particle states are influenced by the extension or the compression of the material in one direction. We have found that under tensile strain, the system remains semiconducting, but the direct band gap of the pristine monolayer turns into an indirect one when strain values overcome 7%. On the other hand, compressive strain has a distinct effect on both strain directions. For values of strain up to −3%, the gap slightly reduces in magnitude but stays direct. By increasing strain, the band gap becomes indirect but it reduces much faster when strain is along the zigzag direction: a semiconductor-to-metal transition occurs when the strain along the zigzag direction is larger than 7%. In contrast, the material remains semiconducting when strain is applied along the armchair direction. The charge distribution is selectively dependent on the strain direction. The bond polarization increases more considerably when strain is along the zigzag direction rather than along the armchair one, as demonstrated through the analysis of the partial charges and the wave functions of the frontier bands.

These results provide insight into the electronic properties of MoTe_2_ under different types of strain and directions. While the analysis presented here is focused only on flat monolayers subject to a homogeneous amount of uniaxial strain, our findings offer an important point of comparison for the study of MoTe_2_ in particular, and TMDC monolayers more generally, being subject to mechanical deformations that give rise to curved or rippled structures [28,29,91]. Dedicated work focused on these systems will be the subject of future publications. Likewise, we expect this study to offer valuable insight for predicting and understanding the electronic and optical responses of heterostructures formed by stacked TMDCs, as well as by hybrids with halide perovskites, which are known to enhance the electrical and optical characteristics of the TMDCs [92,93]. To explore these systems in which one or more layers are likely strained, the knowledge gained from this work is expected to set the stage for additional research specifically dedicated to charge transport and dynamics.

## Data Availability

The data that support the findings of this study are available at the Zenodo database with the following: https://doi.org/10.5281/zenodo.8221339, accessed on 26 August 2023.

## Supplementary Materials

The following supporting information can be downloaded at: https://www.mdpi.com/article/10.3390/nano13202740/s1. References [94,95,96] are cited in the supplementary materials.
