# Supplementary material for: Strained Monolayer MoTe2 as a Photon Absorber in the Telecom Range
Source: Nanomaterials (Basel). 2023 Oct 10;13(20):2740. doi: 10.3390/nano13202740 (PMC10608843; doi:10.3390/nano13202740)
Supplement: Supplementary file 1 [file nanomaterials-13-02740-s001.zip › nanomaterials-2605779-supplementary.pdf]

Supporting Information

Strained MoTe2 Monolayer as Photon Absorber in the Telecom Range

Muhammad Sufyan Ramzan[1], Caterina Cocchi[1,2]

[1]Institut für Physik, Carl von Ossietzky Universität, 26129 Oldenburg, Germany.

[2]Center for Nanoscale Dynamics (CeNaD), Carl von Ossietzky Universität, 26129, Oldenburg, Germany.

Correspondence to: muhammad.sufyan.ramzan@uni-oldenburg.de,

caterina.cocchi@uni-oldenburg.de

In the Supporting Information, we provide phonon band structures and HSE06 band gaps of selected strained $MoTe_2$ monolayers. We also display the electronic band structures computed, at the PBE level, for all considered strained configurations of $MoTe_2$, including additional analysis of the wave function distribution with refined computational parameters. Information on the lowest energy excitations computed for selected strained $MoTe_2$ monolayers is also reported

S1. Dynamical Stability of Strained $MoTe_2$ Monolayers

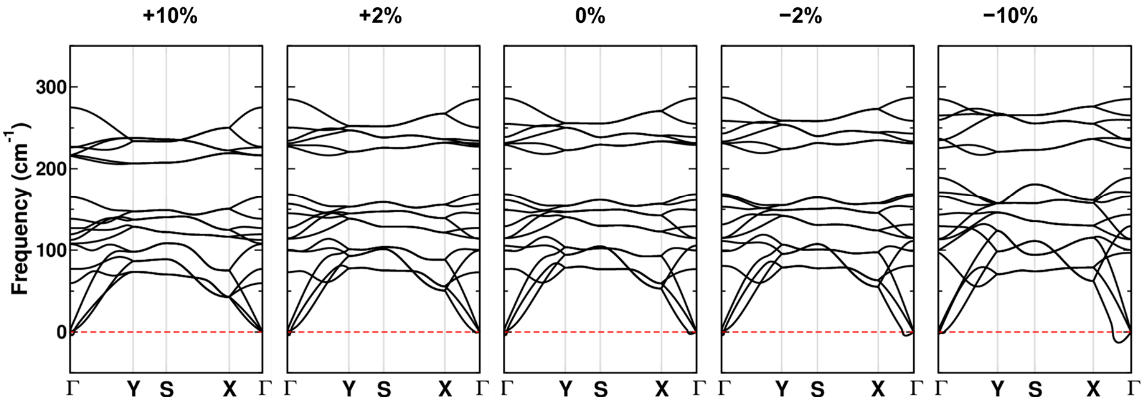

Figure S1. Phonon dispersion of monolayer $MoTe_2$ under tensile (positive values) and compressive strain (negative values) in the armchair direction. 0% refers to the unstrained configuration.

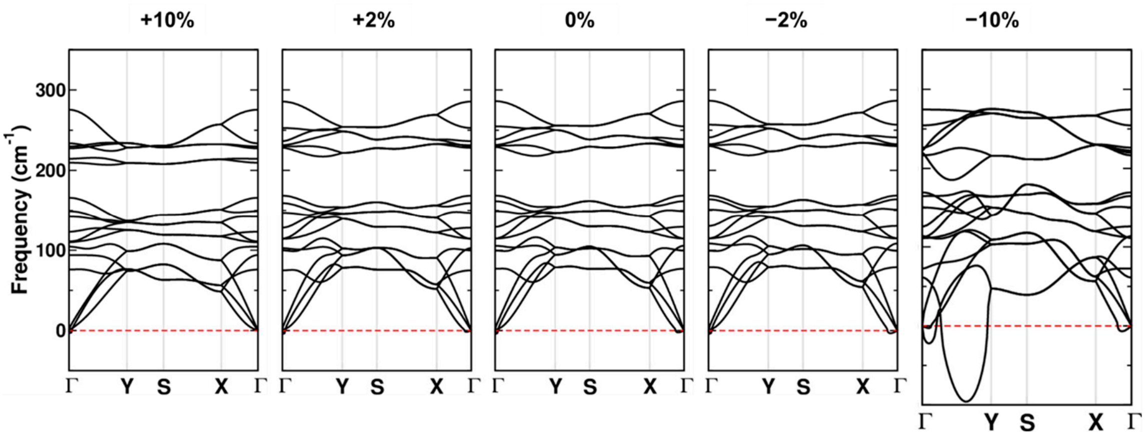

Figure S2. Phonon dispersion of monolayer $MoTe_2$ under tensile (positive values) and compressive strain (negative values) in the zigzag direction. 0% refers to the unstrained configuration.

We investigated the dynamical stability of the strained monolayer $MoTe_2$ by calculating the phonon dispersion of selected structures including the unstrained system for reference as well as sheets with |2|% strain, corresponding to medium-low deformations, and |10|% strain, representing extreme deformations. The results reported in **Figure S1** and **Figure S2** for strain applied in the armchair and zigzag direction, respectively, indicate that the considered configurations are dynamically stable. A few negative frequencies around the **Γ** point appear under −10% strain in the armchair direction (**Figure S1,** rightmost panel) which we ascribe to numerical issues related to the supercell size as discussed for other two-dimensional materials [1,2]. Dynamical instabilities with a physical origin are seen upon −10% strain in the zigzag direction (**Figure S2,** rightmost panel) in the form of negative phonon branches between Γ and Y. This reciprocal-space direction corresponds to the strained axis in the real space. This phonon instability may be considered an indicator of a phase transformation occurring under very large strain, as previously discussed in the context of perovskites [3,4] and 2H – 1T' phase transition in monolayer $MoTe_2$[5–9]. It is worth mentioning that these studies are focused on the metallic 1T phase of $MoTe_2$ and, hence, account for several additional stimuli, such as doping and/or temperature, alongside strain. Dedicated studies on dynamical properties of

monolayer $MoTe_2$ under extreme deformations, which go beyond the scope of the present work, are needed to shed light on this feature.

S2 Band-gap calculations with HSE06

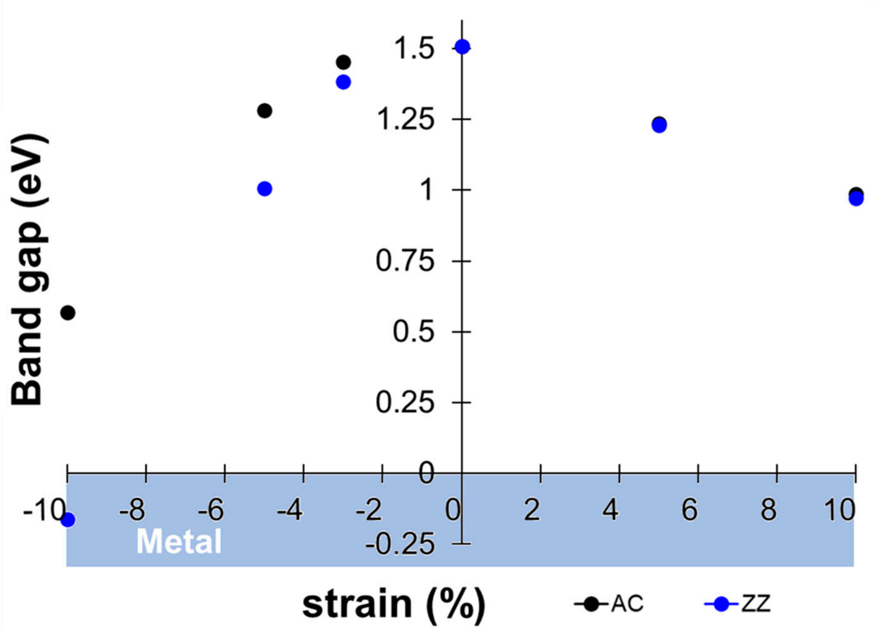

Figure S3. Electronic band gap of MoTe2 computed from HSE06 as a function of strain applied along the armchair (AC, black) and zigzag (ZZ, blue) directions.

S3 Band Structures of Strained $MoTe_2$ Monolayer

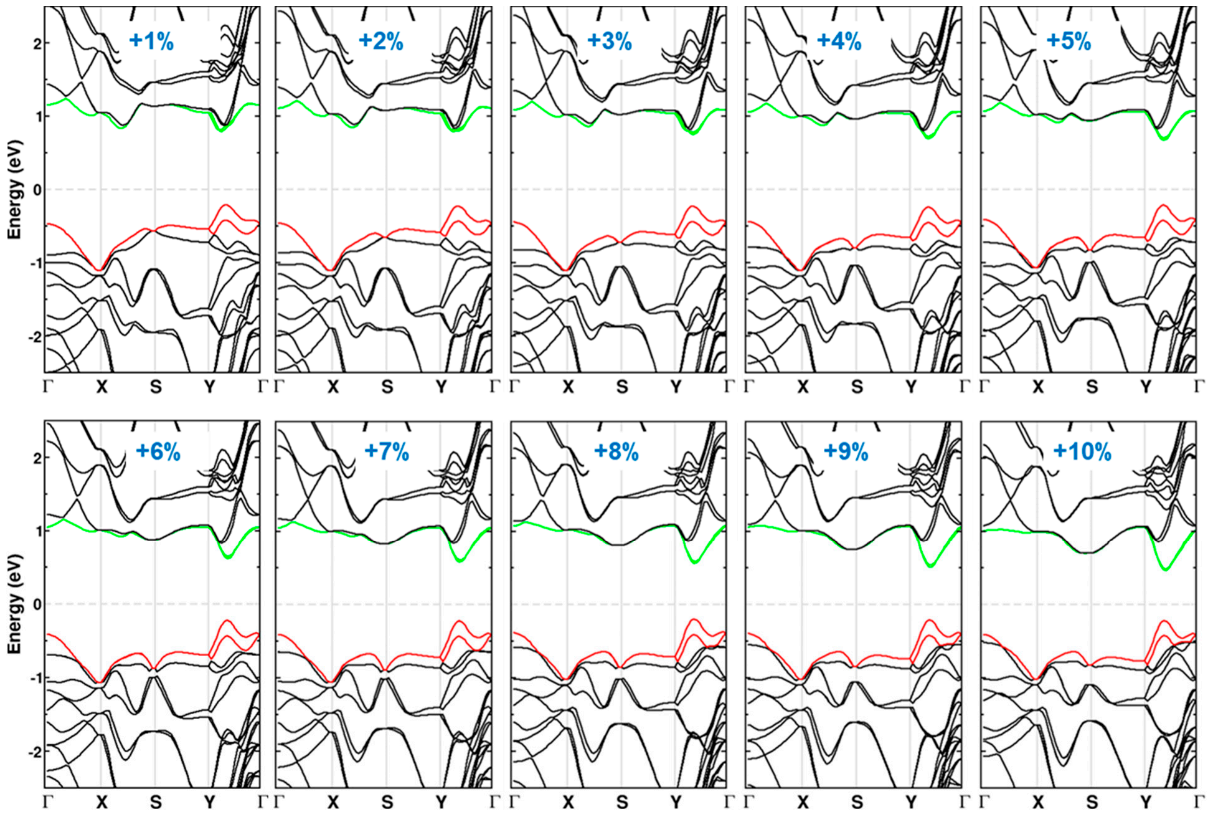

Figure S4. Electronic structures of monolayer $MoTe_2$ calculated from PBE under the indicated values of tensile strain along the armchair direction. The highest valence bands and the lowest conduction bands are highlighted in red and green, respectively. The Fermi level in the mid-gap is marked by a dashed grey line and set to zero.

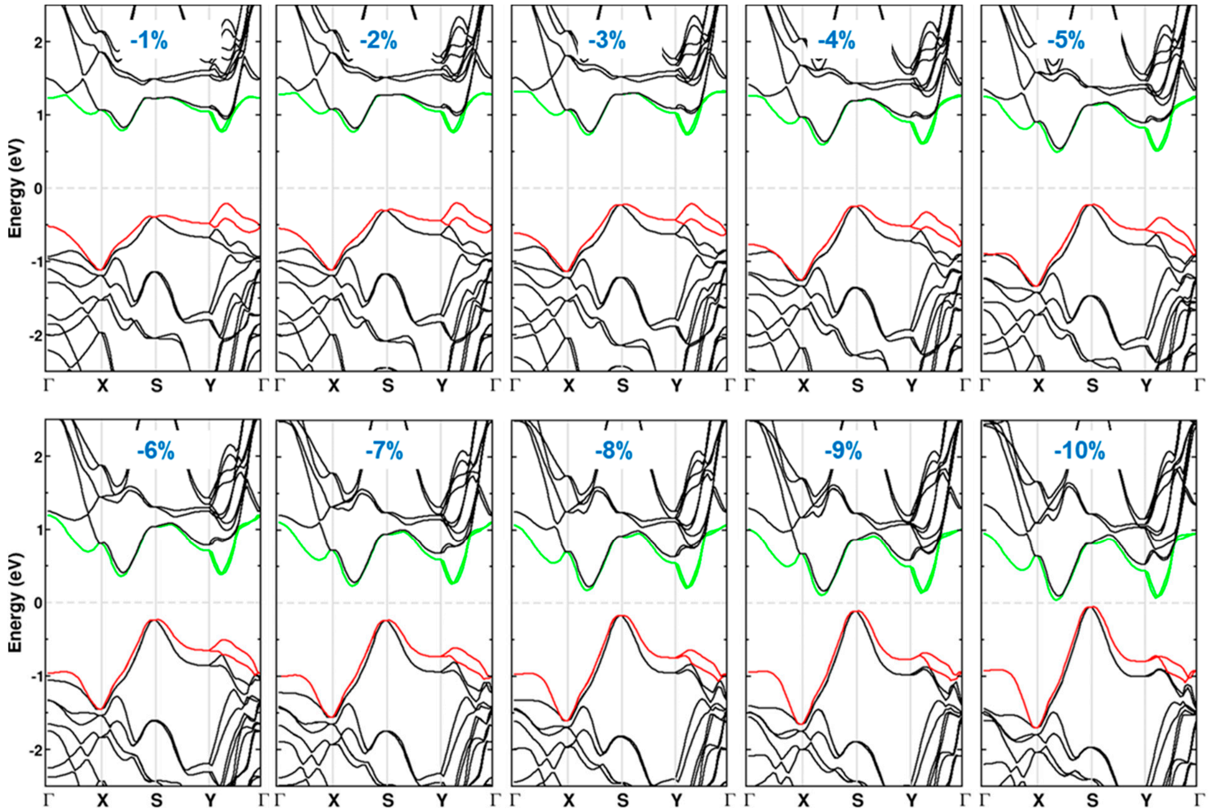

Figure S5. Electronic structures of monolayer $MoTe_2$ calculated from PBE under the indicated values of compressive strain along the armchair direction. The highest valence bands and the lowest conduction bands are highlighted in red and green, respectively. The Fermi level in the mid-gap is marked by a dashed grey line and set to zero.

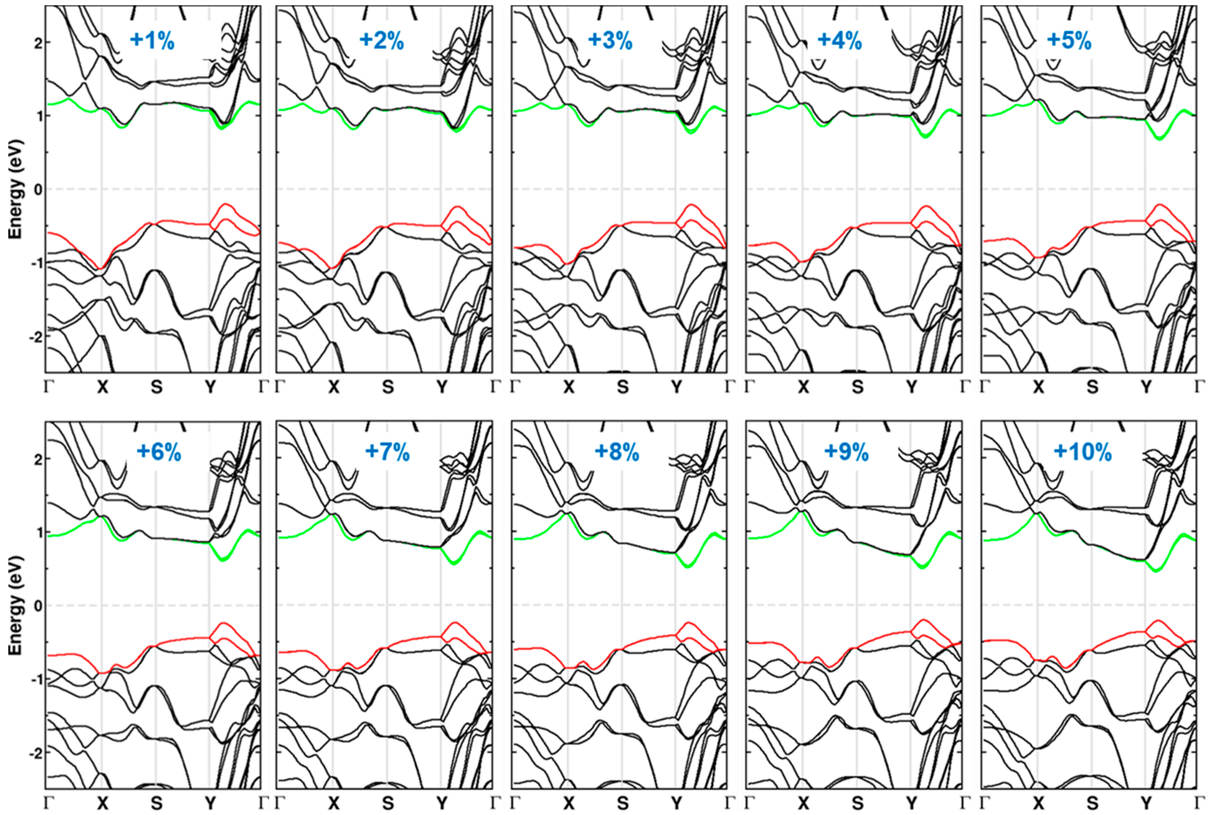

Figure S6. Electronic structures of monolayer $MoTe_2$ calculated from PBE under the indicated values of tensile strain along the zigzag direction. The highest valence bands and the lowest conduction bands are highlighted in red and green, respectively. The Fermi level in the mid-gap is marked by a dashed grey line and set to zero.

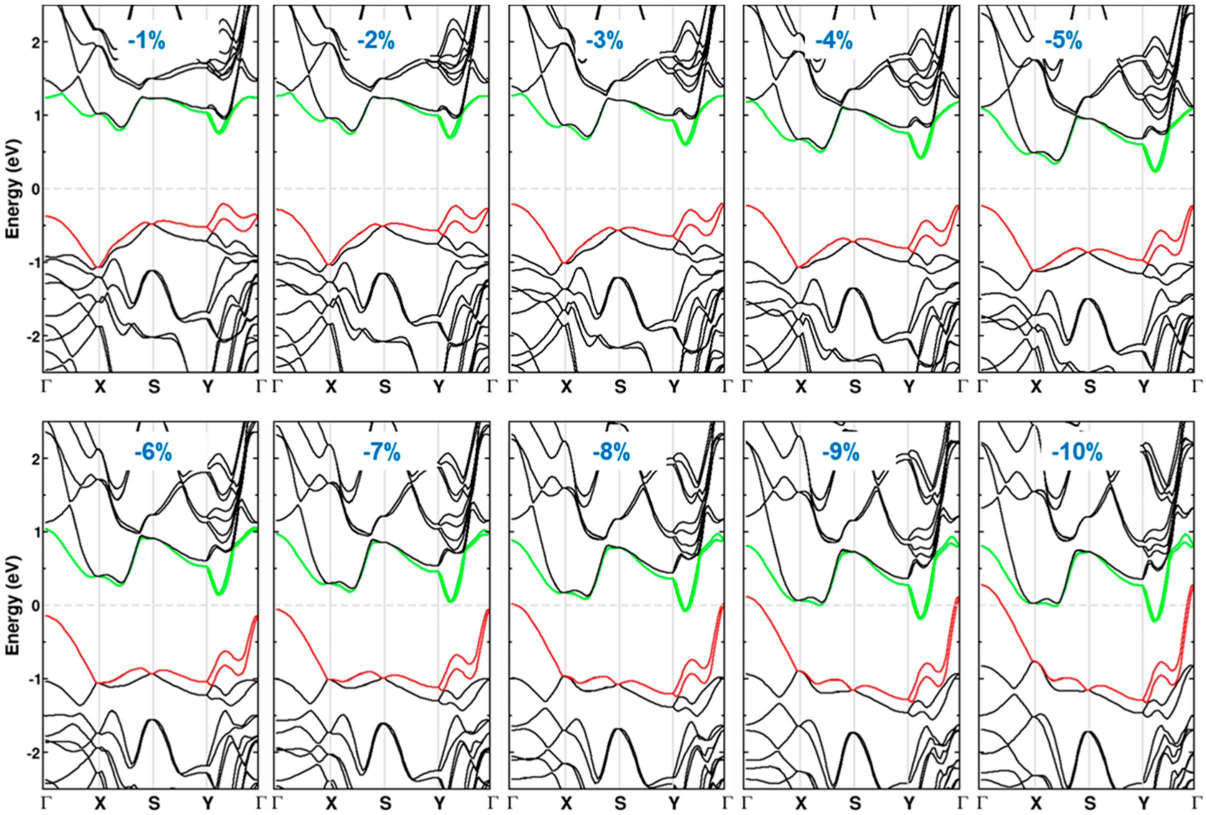

Figure S7. Electronic structures of monolayer $MoTe_2$ calculated from PBE under the indicated values of compressive strain along the zigzag direction. The highest valence bands and the lowest conduction bands are highlighted in red and green, respectively. The Fermi level in the mid-gap is marked by a dashed grey line and set to zero.

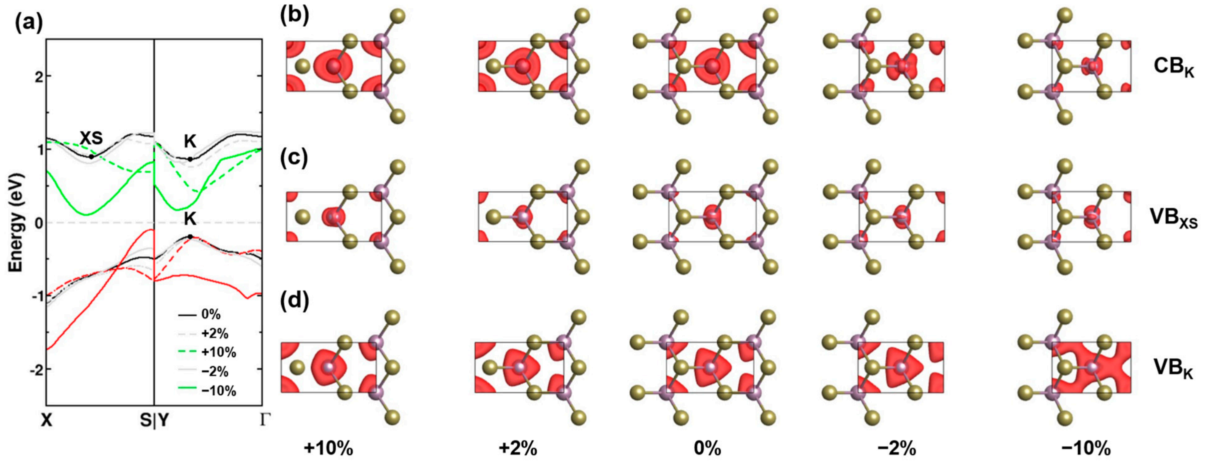

Figure S8. (a) Electronic band structure and (b) – (d) the real space visualization of the probability density of KS states at selected points in the reciprocal space under the specified values of compressive and tensile strain along the armchair direction. These results shown in panel (a) are obtained with a number of **k**-points between X and S as well as between Y and Γ that is double with respect to their counterparts in Fig. 4 in the main text. By comparison with Fig. 4 one does not notice any significant difference that may point to artifacts due to k-point undersampling. The isosurfaces in panels (b) – (d) are plotted with an isovalue of 0.0035.

S4 Optical Excitations in Strained Monolayer $MoTe_2$

Table S1. Energy of the first excitation (E_{S1}), of the first bright excitation (E_{bright}) with its index (different from 1 if the first one is dark), GW band gap (Δ_{GW}) and binding energy (B.E.) of the first bright excitation computed as the difference between the GW gap (Δ_{GW}) and its energy (E_{bright}), for ML $MoTe_2$ under selected values of strain along the armchair (AC) and zigzag (ZZ) directions. Results for the unstrained material are

also provided. All energy values are in eV. It is worth noting that upon large values of applied strain, the first exciton becomes bright.

System	E_{S1}	E_{bright}	index	Δ_{GW}	B.E.
**AC (-2%)**	1.107	1.380	13th	1.787	0.407
**AC (-10%)**	0.984	0.984	1st	1.382	0.398
**AC (+2%)**	1.137	1.170	5th	1.587	0.417
**AC (+10%)**	0.814	0.814	1st	1.191	0.377
**Unstrained (0%)**	1.122	1.278	5th	1.652	0.374
**ZZ (-2%)**	1.070	1.381	21st	1.790	0.409
**ZZ (-7%)**	0.929	1.120	5th	1.512	0.392
**ZZ (+2%)**	1.169	1.169	1st	1.564	0.395
**ZZ (+10%)**	0.785	0.785	1st	1.177	0.392

References

[1] Cheng Y C, Zhu Z Y, Tahir M and Schwingenschlögl U 2013 Spin-orbit–induced spin splittings in polar transition metal dichalcogenide monolayers *EPL* **102** 57001

[2] Ramzan M S, Bacic V, Jing Y and Kuc A 2019 Electronic Properties of a New Family of Layered Materials from Groups 14 and 15: First-Principles Simulations *J. Phys. Chem. C* **123** 25470–6

[3] Lee J H, Delaney K T, Bousquet E, Spaldin N A and Rabe K M 2013 Strong coupling of Jahn-Teller distortion to oxygen-octahedron rotation and functional properties in epitaxially strained orthorhombic LaMnO 3 *Phys. Rev. B* **88** 174426

[4] Garcia-Castro A C, Romero A H and Bousquet E 2016 Strain-Engineered Multiferroicity in P n m a NaMnF 3 Fluoroperovskite *Phys. Rev. Lett.* **116** 117202

[5] Duerloo K-A N, Li Y and Reed E J 2014 Structural phase transitions in two-dimensional Mo- and W-dichalcogenide monolayers *Nat Commun* **5** 4214

[6] Huang H H, Fan X, Singh D J, Chen H, Jiang Q and Zheng W T 2016 Controlling phase transition for single-layer MTe_2 (M = Mo and W): modulation of the potential barrier under strain *Phys. Chem. Chem. Phys.* **18** 4086–94

[7] Song S, Keum D H, Cho S, Perello D, Kim Y and Lee Y H 2016 Room Temperature Semiconductor–Metal Transition of MoTe2 Thin Films Engineered by Strain *Nano Lett.* **16** 188–93

[8] Wang Y, Xiao J, Zhu H, Li Y, Alsaid Y, Fong K Y, Zhou Y, Wang S, Shi W, Wang Y, Zettl A, Reed E J and Zhang X 2017 Structural phase transition in monolayer MoTe2 driven by electrostatic doping *Nature* **550** 487–91

[9] Zhao Y, Li Y, He S and Ma F 2021 Semiconductor-semimetal transition of MoTe2 monolayer modulated by charge-injection and strain engineering *Chemical Physics Letters* **770** 138473
